# Supplementary material for: Active and passive touch are differentially represented in the mouse somatosensory thalamus
Source: PLoS Biol. 2025 Apr 8;23(4):e3003108. doi: 10.1371/journal.pbio.3003108 (PMC11978071; doi:10.1371/journal.pbio.3003108)
Supplement: Attachment — Submitted filename: Active and passive touch in thalamus_ Response to reviewers.pdf [file pbio.3003108.s014.pdf]

Reviewer #1

(Scott Pluta): Sumser et al. show that neurons in the POm region of the thalamus rapidly adapt to active touch but respond reliably to passive whisker stimulation with an air puff. This challenges the previous hypothesis that the POm is more strongly driven by active touch. Some of the other results of the paper are replications of previous publications: Petty…Bruno, 2021; Moore…Kleinfeld, 2015; Ahissar, Sosnik, Haidarliu, 2000. Nonetheless, the primary focus of this paper is on passive vs. active touch, which sets it apart.

Response: Thank you for your in-depth and scholarly review and the helpful suggestions to improve our paper.

Below are a few areas of concern, which if addressed, will enhance our understanding of the results.

From figure 1, sometimes mice whisk after experiencing an air puff and sometimes they don't. Since POm spiking increases with whisking/arousal, how much of the puff response can be explained by puff-induced whisking? Please compare POm activity between puff events that did and did not elicit whisking. I realize that the presence/absence of the behavioral response could correlate with stimulus adaptation, making data selection criteria difficult.

Response: As suggested, we compared POm/VPM activity between puff events that did and did not induce whisking. This comparison shows very similar responses regardless if puffs induced or did not induce whisking, albeit whisking-inducing air puffs lead to slightly larger modulation of VPM (see below). We therefore retained our original data selection criteria, i.e. keeping response trials pooled, regardless whether they induced or did not induce whisking.

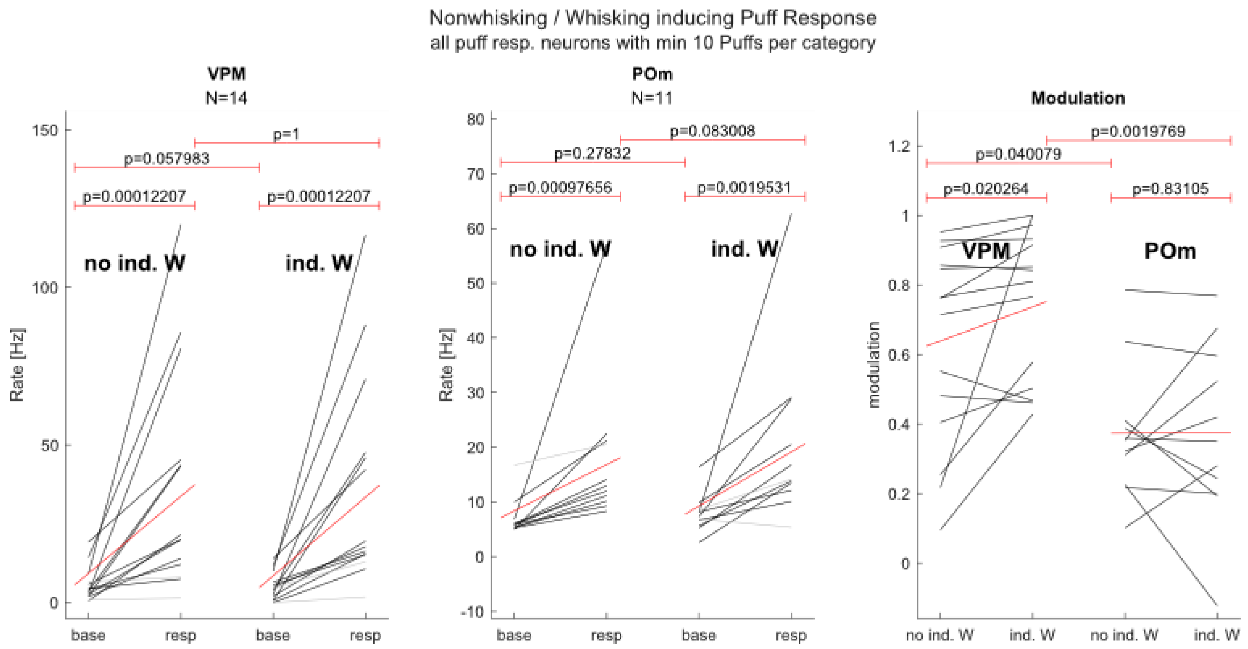

A) VPM spike rates during baseline and after whisker deflection via puff. Baseline: 50 ms windows before, response (resp): 50 ms after deflection. Individual neurons (black: rate increase $p < 0.05$, gray: non-significant increase) and

population means (red). Trials were spit into trials that did not induce whisking (no ind. W) and trials that induced whisking (ind. W).
(B) Same as A but for POm.
(C) Spike rate modulation by puff compared between trials that did not induce whisking (no ind. W) and trials that induced whisking (ind. W). Individual neurons (black) and population means (red).

P values between conditions: two-sided Wilcoxon signed-rank test; between neuron populations: two-sided Wilcoxon ranked sum test; Individual neuron comparison: one-sided Wilcoxon signed rank test.

Since the mechanism of POm not responding to active touch is sensory adaptation, it would be useful to show this effect more explicitly, rather than having it buried in one figure panel: 4I. Perhaps it would be useful to show the POm response to the first touch in the bout of active touch, to reveal the "least adapted" response.

Response: We followed up on this suggestion, by adding a new analysis on the sensory adaptation during whisking bouts. As suggested, we compared the first touch responses in the bout to the remaining touch responses in the bout. VPM neurons on average responded to the first and remaining touches, while POm neurons only responded to the first touch in a bout and were unable to maintain responsiveness to later touches in a bout. Together with the kinematic- and whisking state analysis (Figs. 3 & 4), we conclude that the relative insensitivity of POm to touch is the result of sensory adaptation during whisking and stimulus repetition during whisking bouts, rather than kinematic differences between touch and puff. Thank you for the suggestion to show the role of intra-bout adaptation more directly. We added the results to the new panels Fig. 4J:

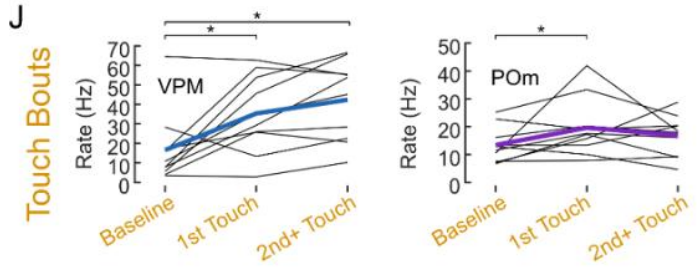

(4J) Responses to 1st vs. subsequent (2nd+) touches within a touch bout. Individual neurons (black) and population means for VPM (blue, left, n = 10) and POm (purple, right, n = 10). Baseline: 50 ms windows before 1st touch in bout, touch: 50 ms after. Only neurons with at least 10 touches in each condition were included.

Does the amount of time the animal spends free-whisking before puff stimulation influence the sensory response? If so, then that would suggest the POm is updating its sensorimotor prediction via self-motion (no touch), and the puff stimulus (externally generated touch) violates that prediction.

Response: Based on this important comment, we revisited the analyses of the relationship between puff responses and whisking state. We account now for a larger window (500 ms) as a proxy for free-whisking preceding the puff stimulation. We define ‘whisking’ as mean whisking amplitudes of >3° in the 500 ms before the puff. With this stricter approach we find that whisking in air before puff stimulation reduces puff responses in both VPM and POm (revised Fig. 3 E-I), consistent with a whisking-mediated sensory adaptation. Interestingly, yet consistent with our original conclusions, the whisking state alone can still not fully explain the insensitivity of POm to touch, as revealed by comparing ‘whisking’ puff responses to touch responses. Here, the modulation by touch remains close

to zero, while modulation by ‘whisking’ puff is significantly stronger (new panel Fig. 3J). We conclude that the touch insensitivity of POm is the result of sensory adaptation which comes about both by whisking and repetitive touch events during a touch-bout (new Fig. 4J). This further supports our original conclusion that POm receives touch information from the cortex and ties in with previous work demonstrating the strongly depressing connection between cortical L5 and POm, which could function as a detector of rare/salient events. We updated all relevant figures and numbers according to the revised definition of whisking and refined our conclusions accordingly at several places (abstract, results (lines 174-193) and discussion (lines 373-383)).

Do you know if the POm neurons in your study are from the first (rostral) and/or second order region? Given the differences in the inputs and outputs, this could explain why some of your POm neurons have a short latency puff response, while others have a long latency response. See https://www.nature.com/articles/s41467-020-17087-7. Given that in Fig. 5F, some long latency responses persist during S1 deactivation, then we can conclude that the long latency responsive neurons are not from the second order Po?

Response: El-Boustani et al. 2020 describe two distinct POm input/output pathways: 1) Sp5i -> POm-FO -> wS2:L4; and 2) Ctx:L5&6 -> POm-HO -> Ctx:L1&5A. They locate POm-FO in rostral POm (approximately between -1 to -2 mm CR) and POm-HO in caudal POm (approximately between -2 to -2.5 mm CR). Axonal calcium imaging suggested that POm-FO and POm-HO respond with similar latency to whisker stimulation. It is thus difficult to infer whether POm neurons in our study belong to POm-FO or POm-HO solely based on response latencies. However, as the reviewer points out, El-Boustani et al. 2020 suggest that POm-FO but not POm-HO neurons receive tactile inputs from the periphery. Thus, our BC suppression experiments (assuming complete suppression of BC) could in principle isolate POm-FO neurons by their persistent whisker-responsivity during BC suppression. As the reviewer points out, our Fig. 5F shows a subset of neurons which maintain their responses during BC suppression. We checked the caudo-rostral locations of these neurons (red shaded neurons in the figure below) and found that they extend across the POm-FO and POm-HO regions á la El-Boustani, while being located close to the approximate dividing line of -2 mm. We would like to note that this analysis is limited because of the small sample size of our data set and because BC suppression may not have been complete.

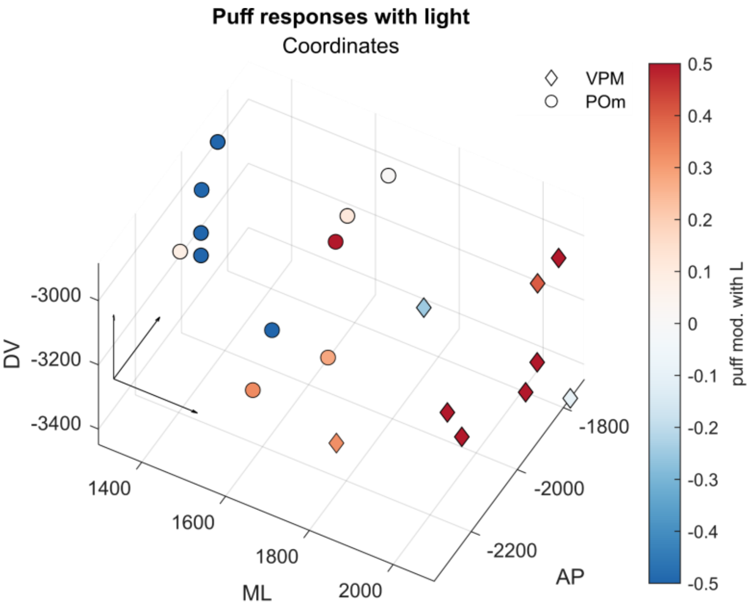

Reconstructed neuron locations in VPM (diamonds) and POm (circles), color-coded with puff modulation upon air puffs during cortical optogenetic inhibition.

Since VPM active touch responses are smaller than VPM puff responses at all strengths (curvature/acceleration), then is VPM also optimized for encoding passive stimulation? Or can this simply be explained by stimulus repetition rate? Figure 4H suggests that repetition rate is not the mechanism for this difference, correct?

Response: Indeed, VPM neurons show weaker responses to active touches, compared to puffs. This holds in general true for all state and kinematic comparisons we analyzed. The effect can mostly be explained by the elevated base rates during whisking (Fig 2C, D) with concomitantly similar response rates (see panel B below). Modulations by whisking puffs are however still stronger than in active touches (now more explicitly shown in new panel Fig 3J), supporting a higher sensitivity of VPM for passive deflections. Stimulus interval does play some role in VPM, such that long interval air puffs elicit stronger responses than short intervals (Fig. 4G). Long interval air puff responses are however still stronger than long interval active touches, suggesting a mechanism beyond repetition rate (Fig 4H). VPM neurons are modulated by whisking phase (Fig 3D), which could account for such response differences. Indeed, air puffs occurring during the protraction phase (Supplementary Fig 3I) elicit comparable modulation as with touches. As a caveat however, phase analysis can be affected by the deflection itself. This point is now elaborated in the discussion (lines 412-417).

It may be useful to look at raw increases in spike rate (relative to baseline spike rate), since modulation indices get smaller as the denominator (baseline spike rate) increases.

Response: We agree that using modulation as a metric can be influenced by the denominator (baseline spike rate). As suggested, we compared the modulation metrics to raw spike rates (absolute, relative to baseline and fold-change). As you see below, there are no qualitative differences between the measures.

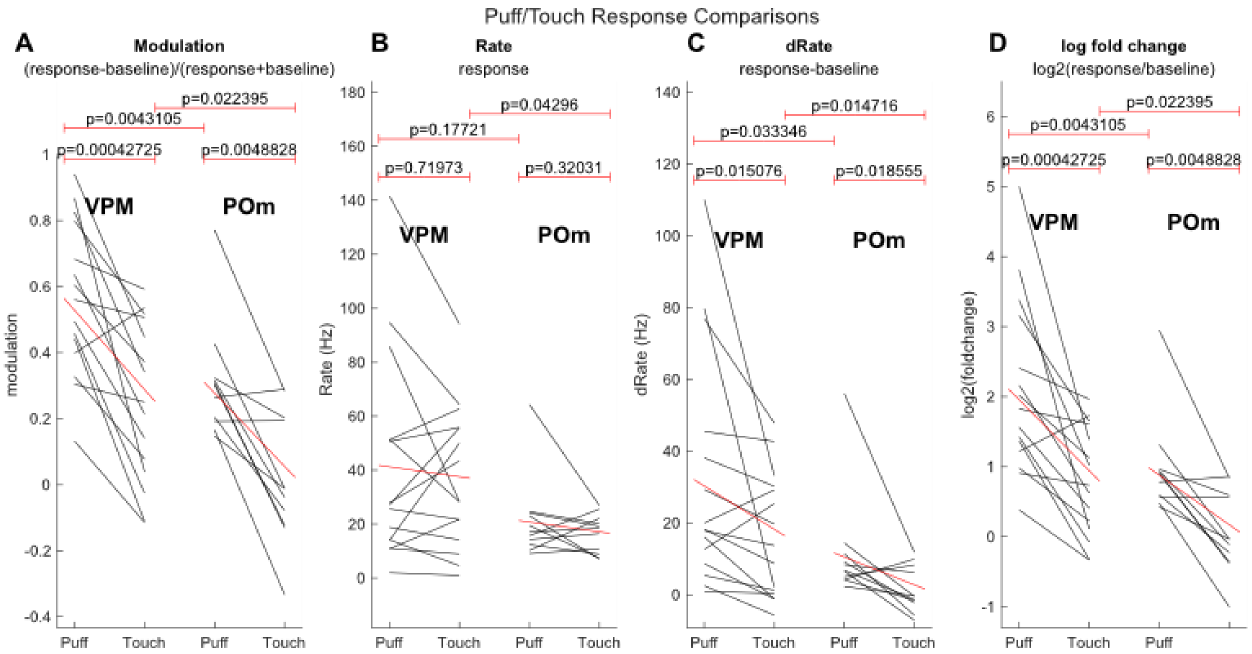

A) Modulation comparison between puff and touch in VPM and POm neurons. Baseline: 50 ms windows before, response (resp): 50 ms after deflection. Individual neurons (black) and population means (red). Same approach and data as in Fig 2E.
(B) Same as A but using raw response rate as a metric
(C) Same as A but using raw rate difference as a metric
(D) Same as A but using log rate fold change as a metric

Technical concern:
Is it possible that the air puff was hitting additional whiskers? Since POm neurons have multi-whisker receptive fields, multi-whisker stimulation could activate them better.

Response: Indeed, POm and some VPM neurons have multi-whisker receptive fields. Therefore, to enable precise response timing analyses and to avoid across-whisker-adaptation during active touches, we deliberately cut all whiskers except one and the air puff tube opening was carefully positioned to only deflect the spared whisker (C1 or C2). The recording site for the spared whisker was mapped in a preceding anesthetized experiment one day before the awake experiment. We now better explain this in the results (line 83) and describe the mapping process in the methods (Craniotomy surgery and receptive field mapping).

Reviewer #2:

This article answers key questions about the long-mysterious role of PO thalamus in sensory coding of active touch. The authors successfully undertake technically challenging experiments that have not previously been possible, resulting in the resolution of many years of apparently conflicting findings in the field. Specifically, the results first replicate past findings on the role of VPM and PO in signaling vibrissa self-motion and tactile responses. They then clarify that that these sensory response properties arise from a strong dependence of PO thalamus on ongoing cortical activity in alert, actively whisking animals. This study is expertly carried-out and provides novel, important

information to the field. Much of the past work on this topic has been previously published in PLOS Biology, making this a fitting journal for publishing these findings.

Response: We thank the reviewer for the encouraging review and the support for timely publication of the results, which we highly appreciate.

Reviewer #3:

In this study, Sumser and colleagues investigate the potential role of first-order vs higher-order thalamic sensory relay using the rodent whisker somatosensory system as a model. This system is ideal to study active vs passive modes of touching since rodents are capable of actively moving their whiskers while they explore their environment. Previous studies have shown contrasting results concerning the responses of POm neurons to various whisker sensory stimuli.
Here, the authors perform juxtacellular recordings in awake animals and find that responses to passive deflections in POm neurons are affected by cortical feedback inactivation, indicating that they rely on the cortex to carry information about the salience of a sensory signal, while most POm neurons do not respond to active touch, when the salience might be lessened. The manuscript is well laid out, however, there is a lack of clarity in the description of some of the analysis in terms of what is included vs excluded making it hard to interpret at times and I would therefore like the authors to address the following points.

Response: Thank you for your in-depth and scholarly review and the helpful suggestions to improve our paper.

Major Points:

1) I fail to understand how such a low number of neurons from the total number of air-responsive neurons were included for further analysis, especially in the VPM (ie: 15 out of 24 VPM neurons and 11 out of 14 POm neurons), considering the low number (n=10) of minimum active touches required for neurons to be included for further analysis in Figure 2. Considering that mice move their whiskers at around 6-9Hz, it shouldn't take much more than 1 second of whisking for an object positioned in front of the animal to be touched at least ten times. Did the authors have difficulties in getting the mice to whisk? Mice can even further increase their whisking rates during active touch since the object reduced the amplitude of their whisker protractions. In effect, touches can end up being separated by only a few 10ms in previous publications (Petersen lab for example). Did the authors stipulate a minimum time separation for active touches to be included in the analysis presented in the paper so as not to overlap with each other on the PSTH of Figure 2B for example? If so, please state it in the Methods.

Response: We did not enforce a minimum time separation between touches in the data selection criteria. The touch object was positioned in a way that only large amplitude whisks could elicit touches, resulting in a relatively low number of touches (median touch rate: 0.5 Hz, interquartile range: 0.4 Hz, across included recordings) and relatively long intervals (median: 99 ms, interquartile range 131 ms, across included recordings). As pointed out, touches within a whisking bout can in principle accumulate quickly. In our hands mice often touched the object a few times only and then spent long periods without spontaneous large amplitude whisking (e.g. whisking at >15° amplitude only in 7% of

the recording time, across included recordings). Our approach was thus a bit different compared to previous studies in which the touch objects were positioned closer to the whisker's resting position, so that the animals had no option but to touch the objects during whisking [1,2]. This likely explains that we had in general fewer touch events (median 126 touches, 3 VPM and 2 POm neurons had 17-50 touches). Furthermore, in each recording, we first determined puff responsiveness with an online analysis and continued recording free whisking, touch and repeated air puff episodes, leading to sometimes losing the cell before a sufficient number of touches could be recorded. This is now clarified in the methods (lines 613-651). In summary, our data exclusion criteria were solely based on the minimum number of recorded touch events.

2) In the methods section, it is stated that "recorded neurons were only included if they showed a significant rate increase in response to air puffs".
It is therefore rather redundant for the authors to state on l.114-116 that "Both VPM and Pom neurons robustly responded to passive deflections."

Response: We removed the sentence.

Later on, the authors state that 9/15 VPM vs 4/11 POm neurons "responded" to active touch. I have not seen in the methods how this was determined in terms of statistics for this subset of cells.

Response: We now better describe this in the methods: "To determine individual neuron's responsiveness to puffs and touches, spike counts in baseline and response windows (each 50 ms) were compared for each trial and significance was estimated with a Wilcoxon signed rank test (one-sided for puff responses to only include neurons with stimulated principal whisker, two-sided for touch responses)."
The statistical test is also mentioned in the legend: "Individual neuron comparison (2C, D): one-sided (Puff) or two-sided (Touch) Wilcoxon signed rank test" (now amended for two-sided tests).

In any case, the final conclusion from figure 2 that "Consistent with a low sensitivity to active touch, POM's firing rate modulation was not significantly different from zero" should be attenuated since 4/11 POm actually DO respond to passive deflections.

Response: The reviewer is correct, we describe this now more accurately: "...POm's firing rate modulation was on the population level not significantly different from zero…"

From Figure 2E, it also appears that one or more of these might even be negatively modulated by air touch. A pie chart of significantly positively or negatively responding VPM and Pom neurons might be more appropriate at this stage of the results rather than the present broad assignment of VPM = responsive to air touch, POm = not responsive to air-touch. I also believe the authors should discuss more this heterogeneity of responses of POm in the discussion. From their results, things do not appear as clear-cut as they sometimes make it out to be, especially in terms of single-cell modulation of activity vs grand average modulation.

Response: As suggested we tested for decreased firing rates using a two-sided test on the data in Fig. 2C-D and included insets showing pie charts showing the heterogeneity. One out of 11 POm

neurons is significantly less active after the touch. Nevertheless, we agree that there is substantial variability within the nuclei and have now discussed this more thoroughly (lines 386-401 and 413-418).

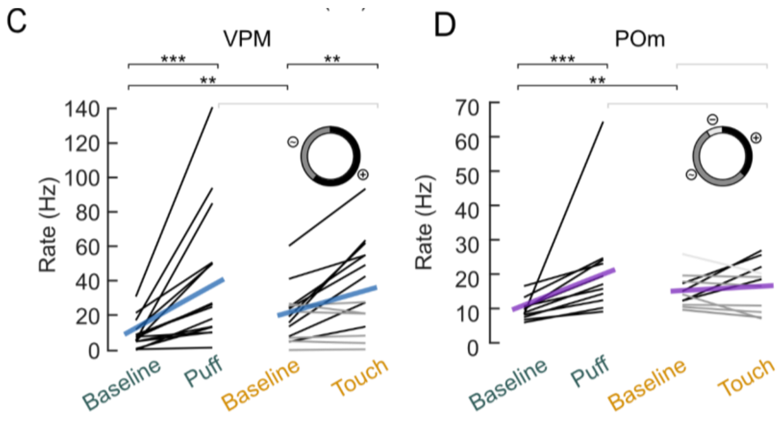

(C) VPM spike rates during baseline and after whisker deflection via Puff or Touch. Baseline: 50 ms windows before, Puff / Touch: 50 ms after deflection. Individual neurons (black: rate increase $p < 0.05$ (9/15 neurons), dark gray: non-significant (6/15 neurons)) and population means (blue). Inset: pie chart of neurons with positive, negative and non-significant responses to active touches.
(D) Same as C but for POm (purple). Rate increase: 4/11 neurons, rate decrease: 1/11 neurons (light gray), non-significant: 6/11 neurons.

3) Free whisking consists of two separate phases, protraction and retraction. Since air puffs are oriented to induce a backward retraction, one would expect some differences in the way neurons respond to air puffs that is dependent upon the protraction/retraction phases of whisking. Did the authors perform such analysis?

Response: We performed this analysis in Supplementary Fig. 3 H&I. While VPM puff responses appear similar across different phase positions, POm puff responses are indeed weaker during protractions, which could contribute to touch/puff response disparity (now added to results (lines 258-261)). Phase computation itself is however affected by the whisker trajectory disturbance of the deflection, limiting interpretation of this finding.

4) In figure 4, it is not clear to me whether air puffs during free whisking are included or not, since Figure 4A seems to indicate very low accelerations during air puffs compared to touches. It appears Q-puffs were included? In which case, the acceleration distributions are not comparable, since the mouse is whisking during active touch. Therefore, tertiles of theses distributions are not similar. It would be preferable to perform the analysis on the basis of the same acceleration distributions (ie: taking into account only W-puffs, and even then, those air puffs that were applied when the mouse was protracting in a similar manner do when it performs active touches). Is there are reason why this was not performed (not enough W-puffs for example)? In any case, this disparity between acceleration distributions should be further discussed.

Response: We included all puff conditions (W-puff and Q-puff) in the analysis of Fig 4 in order to tease apart if detailed differences in the deflection kinematics between puff and touch stimuli can explain their response disparities. With this approach we show (1.) that there are substantial differences in

some of the distributions, as the reviewer points out: For example, active touches typically induce more absolute angular acceleration (Fig 4A) but smaller absolute curvature changes (Fig. 4D) than air puffs. For this reason, we (2.) aimed to roughly match these differences by splitting into tertiles with similar partial distributions each. While response differences across kinematic tertiles and touch vs. puff responses are evident in both VPM and POm, the major point here is that POm neurons do not respond substantially to touches at any tested kinematic ranges. We expanded on this point in the discussion (lines 385-389).

As the reviewer suggests, we did the same analysis with W-Puffs, which only slightly broadens the distribution of puff-induced acceleration (absolute angular velocity difference after and before puffs should not strongly depend on velocity pre-puff), barely changes curvature and interval distributions, and does not change the conclusions:

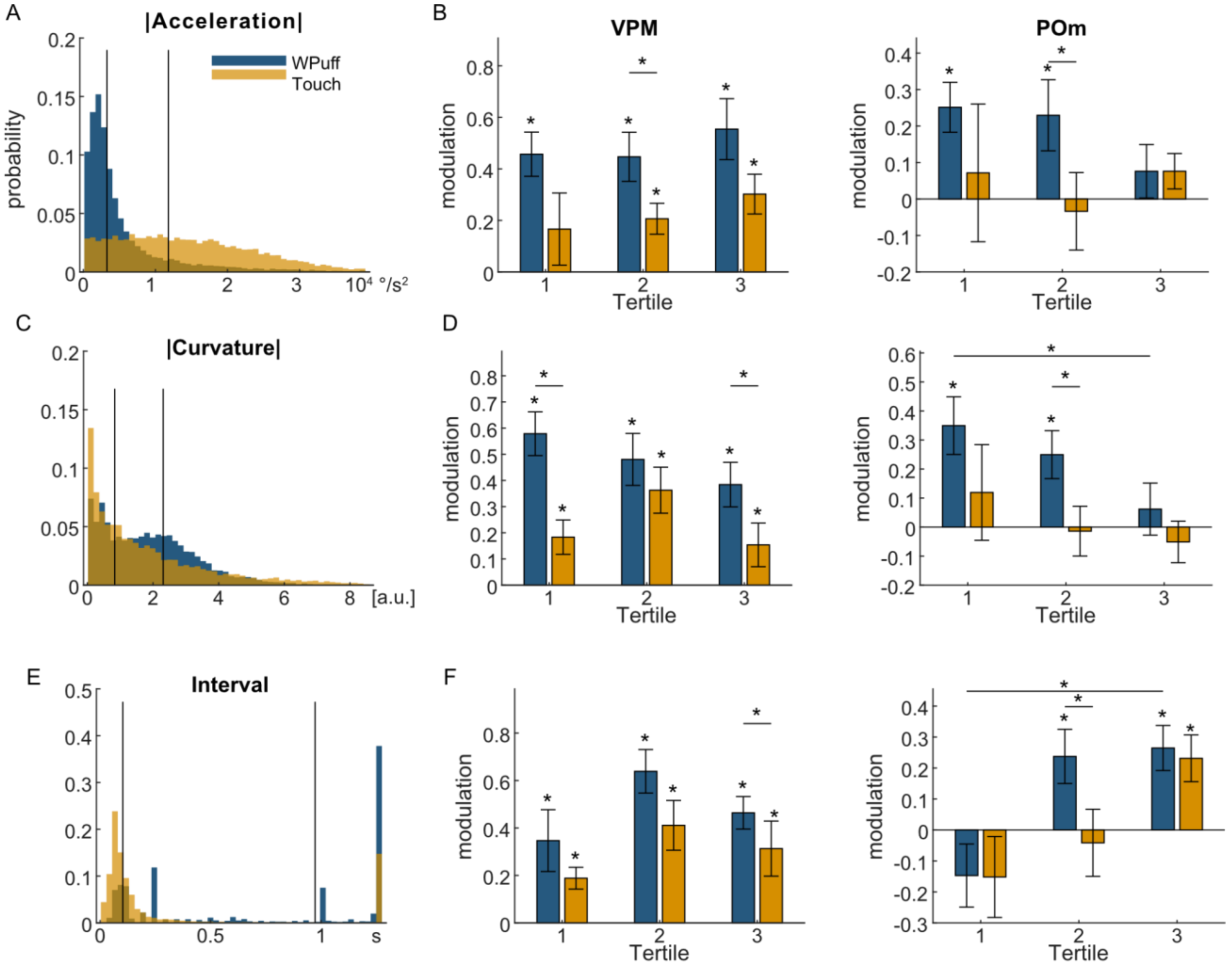

Kinematic matching with air puffs during whisking (WPuff)
(A) Distribution of absolute angular acceleration during (± 25 ms) touch (yellow) and W-puff (blue). Joint distribution divided into acceleration tertiles (vertical black lines). (B) Modulation in response to W-puff (blue) and touch (yellow) split by acceleration tertiles indicated in A for VPM (left) and POm (right). (C-D) Same as in A-B but splitting deflection events into tertiles of whisker curvature following whisker deflection (0 to 25 ms). (E-F) Same as in A-B but splitting deflection events into tertiles based on intervals between whisker deflections.

As suggested, splitting deflection events by whisking phase (during protraction vs. retraction) is shown in Supplementary Figure 3 H, I. Air puffs during protracted whisker angles (Tertile 3 in Supplementary

Figure 3H) and during the protraction phase (Tertile 1 in Supplementary Figure 3I), overlap with the bulk of touch kinematics and indeed lead to low firing rate modulations in POm, which may contribute to the touch/puff disparity. Air puff undersampling in these ranges and deflection effects on the phase computation limit interpretation of this finding, but the possibility is now discussed (lines 258-261).

5) Similarly, active touches are divided into tertiles in Figure 4. But if the minimum number of touches is 10, does this mean that for some cells, a "tertile" consists only of a few touches? Please elaborate on this point, since statistical significance is essential to the conclusions of Figure 4.

Response: Indeed, in rare cases this could lead to only a few trials per tertile in some neurons because mice were whisking freely and thus each recording showed only a part of the kinematic distributions. For this reason we only show modulation-normalized population average analyses in Fig 4, which were pooled across recordings (now clarified in the results (lines 239-241) and methods (lines 761-763)). Restricting the analysis to neurons with a minimum number of 5 touches and puffs in each tertile did not qualitatively change the results but reduced the sampling size substantially (see below). In summary, while VPM is subtly modulated by kinematic parameters, POm is primarily modulated by stimulus interval such that only long event intervals evoke responses in POm.

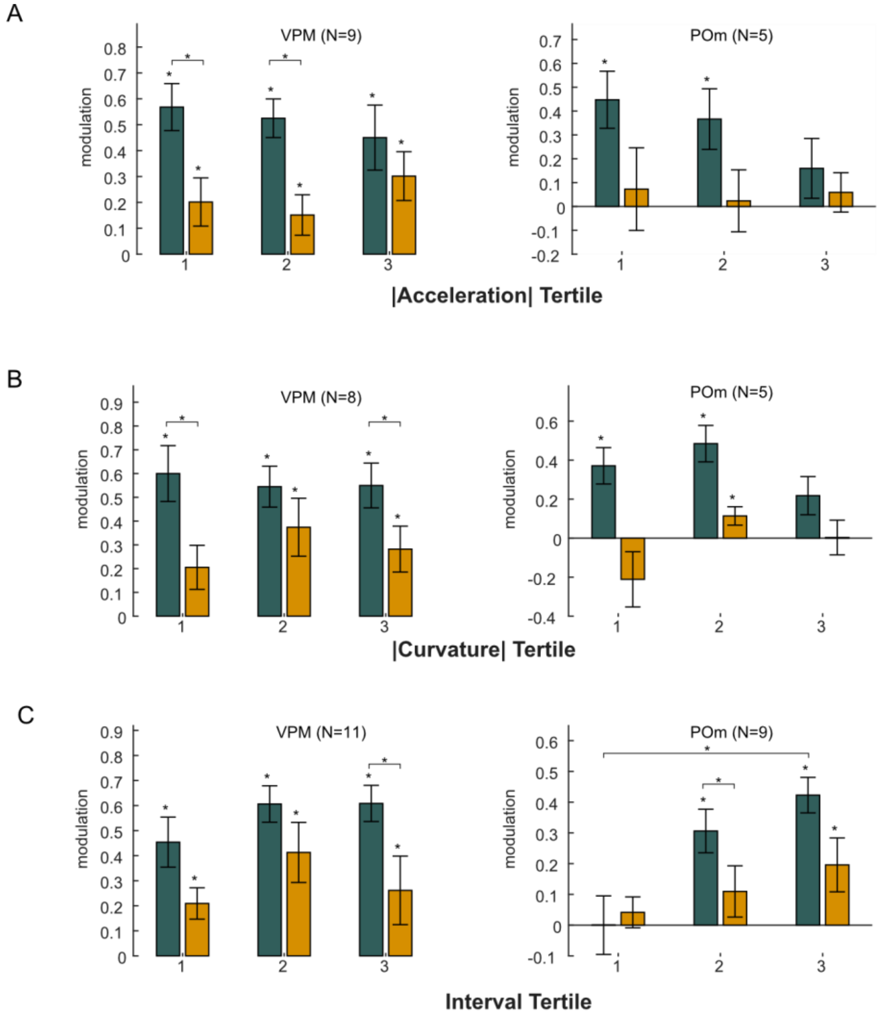

Kinematic and interval modulation in VPm and POm restricted to neurons which had at least 5 stimulus trials in each tertile

(A) Modulation in response to puff (teal) and touch (yellow) split by absolute acceleration tertiles for VPM (left) and POm (right)
(B) as in A for absolute curvature tertiles
(C) as in A for interval tertiles

6) On Figure 5E-F, I find it interesting that VPM responses appear bimodal, and that the late latency component of that response is not affected by cortical inhibition despite the likelihood that this late response (because of its lateness) depends on some form of cortical feedback (from Layer 6). I would like the authors to elaborate on this. Why is this late response unaffected?

Response: The bimodality of VPM whisker responses seen in the population PSTHs in Fig. 5 E&F is most likely the result of pooling over a set of neurons with qualitatively unimodal responses but varying response latencies (see heatmaps and single neuron examples in Fig. 1). The neurons in Fig. 5 are a subset of a larger population of neurons in Figures 1-4 in which the latencies span more continuously. In regards to the late response components in VPM: these have been studied in awake rats and lasted up to 200 ms following the whisker deflection [3]. Hirai et al suggests that whisker responses in the late period are unlikely to arise from ascending inputs, as PrV did not exhibit such late responses. Interestingly, this "recurrent" VPM activity increased after ablations of the barrel cortex. The authors suggest that late VPM responses arise from TRN feedback, which is further "unleashed" after thalamic neurons lose their glutamatergic input from the cortex, which boosts T-type calcium conductances.

7) Similarly, for the POM, I noticed that the neurons that are mainly affected by cortical inactivation are the ones displaying a short latency response (if we look at the heatmaps, it's the ones at the top who's responses disappear, rather than the ones at the bottom of the heatmap). Again, the authors should discuss this dichotomy, possibly in the light of S1 vs S2 feedback and spread of their optogenetic light beam…

Response: The POm neurons where cortical inhibition did not abolish air puff responses were located both in the anterior and posterior part of POm. Putatively, due to topographic L5 projections [4] and nucleus suborganization [5], the efficacy of optogenetic stimulation in the center of the barrel cortex varied. This is now discussed more thoroughly (lines 427-430).

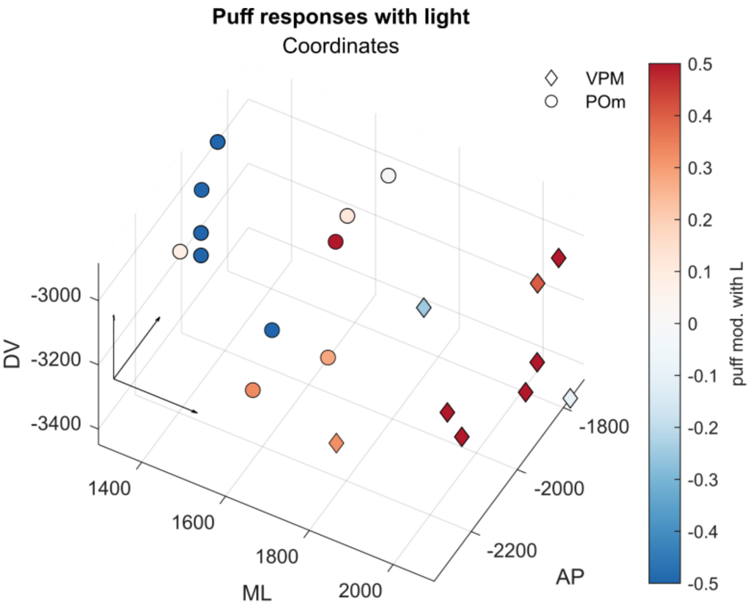

Reconstructed neuron locations in VPM (diamonds) and POm (circles), color-coded with puff modulation upon air puffs during cortical optogenetic inhibition.

Minor points:

1) On line 82, it is mentioned that either C2 or B1 whisker is left intact, but in the methods (l. 489 and l.491), they become C2 or B2. Please correct one or the other.

Response: thank you, it was either C1 or C2, we corrected this.

2) Figure 3: in A-C, we see that about half of the "larger set" of neurons is phase-locked to free whisking. The authors then concentrate on the "air touches >10 subset" for the reminder of the Figure. Does this average of "around half the neurons" still hold for this smaller set?

Response: The proportion of phase locked neurons among all neurons with puff responses was 14/24 VPM neurons and 6/14 POm neurons as reported in the manuscript. The smaller set of neurons that also had a sufficient number of recorded active touches showed similar proportions of significant phase locking: 9/15 VPM neurons and 5/11 POm neurons. Thus, the statement that around half the neurons show whisker phase-locked activity holds true in either analysis. We made this more clear in the results (line 172).

References

1. Yu C, Derdikman D, Haidarliu S, Ahissar E. Parallel thalamic pathways for whisking and touch signals in the rat. PLoS Biol. 2006;4: e124. doi:10.1371/journal.pbio.0040124

2. de Kock CPJ, Pie J, Pieneman AW, Mease RA, Bast A, Guest JM, et al. High-frequency burst spiking in layer 5 thick-tufted pyramids of rat primary somatosensory cortex encodes exploratory touch. Commun Biol. 2021;4: 709. doi:10.1038/s42003-021-02241-8

3. Hirai D, Nakamura KC, Shibata K-I, Tanaka T, Hioki H, Kaneko T, et al. Shaping somatosensory responses in awake rats: cortical modulation of thalamic neurons. Brain Struct Funct. 2018;223: 851–872. doi:10.1007/s00429-017-1522-z

4. Sumser A, Mease RA, Sakmann B, Groh A. Organization and somatotopy of corticothalamic projections from L5B in mouse barrel cortex. Proc Natl Acad Sci U S A. 2017;114: 8853–8858. doi:10.1073/pnas.1704302114

5. El-Boustani S, Sermet BS, Foustoukos G, Oram TB, Yizhar O, Petersen CCH. Anatomically and functionally distinct thalamocortical inputs to primary and secondary mouse whisker somatosensory cortices. Nat Commun. 2020;11: 3342. doi:10.1038/s41467-020-17087-7
